# The crystal structure of the heme *d*_1_ biosynthesis-associated small *c*-type cytochrome NirC reveals mixed oligomeric states *in crystallo*

**DOI:** 10.1107/S2059798320003101

**Published:** 2020-03-25

**Authors:** Thomas Klünemann, Steffi Henke, Wulf Blankenfeldt

**Affiliations:** a Structure and Function of Proteins, Helmholtz Centre for Infection Research, Inhoffenstrasse 7, 38124 Braunschweig, Germany; b Institute for Biochemistry, Biotechnology and Bioinformatics, Technische Universität Braunschweig, Spielmannstrasse 7, 38106 Braunschweig, Germany

**Keywords:** cytochrome *c*, 3D domain swapping, *Pseudomonas aeruginosa*, heme *d*_1_ biosynthesis, NirC

## Abstract

Monoheme *c*-type cytochromes are important electron transporters in all domains of life. They possess a common fold hallmarked by three α-helices that surround a covalently attached heme. An intriguing feature of many monoheme *c*-type cytochromes is their capacity to form oligomers by exchanging at least one of their α-helices, which is often referred to as 3D domain swapping. Here, the crystal structure of NirC, a *c*-type cytochrome co-encoded with other proteins involved in nitrite reduction by the opportunistic pathogen *Pseudomonas aeruginosa*, has been determined. The crystals diffracted anisotropically to a maximum resolution of 2.12 Å (spherical resolution of 2.83 Å) and initial phases were obtained by Fe-SAD phasing, revealing the presence of 11 NirC chains in the asymmetric unit. Surprisingly, these protomers arrange into one monomer and two different types of 3D domain-swapped dimers, one of which shows pronounced asymmetry. While the simultaneous observation of monomers and dimers probably reflects the interplay between the high protein concentration required for crystallization and the structural plasticity of monoheme *c*-type cytochromes, the identification of conserved structural motifs in the monomer together with a comparison with similar proteins may offer new leads to unravel the unknown function of NirC.

## Abstract 2

The crystal structure of the *c*-type cytochrome NirC from *Pseudomonas aeruginosa* has been determined and reveals the simultaneous presence of monomers and 3D domain-swapped dimers in the same asymmetric unit.

## 1. Introduction   

**Figure 1:**
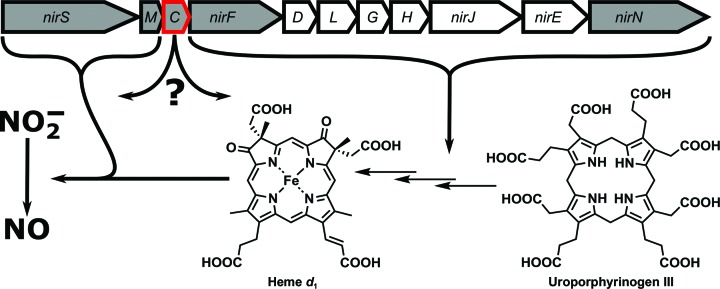
Depiction of the *nir* operon, indicating the function of each gene product in nitrite reduction and heme *d*_1_ biosynthesis. A grey background highlights the periplasmic location of the gene product.

Monoheme *c*-type cytochromes are a subfamily of *c*-type cytochrome proteins and are well known electron transporters in the respiratory chain of organisms in all domains of life. In bacteria, they are also involved in H_2_O_2_ scavenging and act as an electron-entry point for nitrate, nitrite and nitric oxide reductases, which function as terminal oxidases in the respiratory chain under anaerobic conditions (denitrification; Bertini *et al.*, 2006 ▸). The monoheme *c*-type cytochrome domain features three α-helices that surround one *c*-type heme covalently attached to the cysteines of the characteristic sequence motif C*XX*CH *via* thioether bonds. The central iron cation of heme is usually coordinated by a histidine residue at its proximal site, whereas a histidine or, more rarely, a methionine ligates the other side in most cases. Many monoheme *c*-type cytochromes can form dimers and oligomers by exchanging the N- or C-terminal α-helix, which offers attractive protein-engineering opportunities (Hirota *et al.*, 2010 ▸; Hayashi *et al.*, 2012 ▸; Nagao *et al.*, 2015 ▸). We have recently embarked on a structural biology program to investigate the enzymes involved in the biosynthesis of heme *d*_1_, an essential cofactor of the *cd*_1_ nitrite reductase NirS, which catalyses the reduction of nitrite to nitric oxide in, for example, the opportunistic pathogen *Pseudomonas aeruginosa* (Schobert & Jahn, 2010 ▸). The *nir* operon of *P. aeruginosa*, which encodes most of the enzymes involved in heme *d*_1_ biosynthesis in addition to *nirS* itself, also contains open reading frames for two monoheme *c*-type cytochromes termed *nirM* and *nirC* (Fig. 1 ▸; Arai *et al.*, 1990 ▸; Kawasaki *et al.*, 1995 ▸). Whereas NirM is absent in many NirS-producing bacteria and has been assigned to function as an electron transporter for the nitrite reductase (Hasegawa *et al.*, 2001 ▸), orthologs of NirC are found in nearly all NirS producers, but their function is subject to ongoing discussions. It has been demonstrated that NirC from *P. aeruginosa* can act as an electron donor for the nitrite reductase *in vitro* (Hasegawa *et al.*, 2001 ▸). However, the redox potential of NirC from *Paracoccus pantothrophus* has been found not to be optimal for the transport of electrons from the *bc*_1_ complex to nitrite reductase (Hasegawa *et al.*, 2001 ▸; Zajicek *et al.*, 2009 ▸; ). A double knockout of the two better-suited electron transporters cytochrome *c*_550_ and pseudoazurin rendered *P. denitrificans* unable to denitrify despite the presence of NirC (Pearson *et al.*, 2003 ▸). Further, mutants of *P. denitrificans* and *P. fluorescens* that are impaired in their ability to express *nirC* are unable to produce active nitrite reductase owing to a lack of heme *d*_1_. Together, these findings implicate the role of NirC as lying in heme *d*_1_ biosynthesis (Ye *et al.*, 1992 ▸; Boer *et al.*, 1994 ▸); however, this could not be confirmed in *P. aeruginosa* (Hasegawa *et al.*, 2001 ▸). To further investigate NirC, we have determined the crystal structure of heterologously produced *P. aeruginosa* NirC. We find a unique crystal packing that contains monomeric NirC together with two different dimeric forms. To our knowledge, this is the first time that monomers and dimers of a monoheme *c*-type cytochrome have simultaneously been observed in the same asymmetric unit.

## 2. Materials and methods   

### 2.1. Macromolecule production   

The gene encoding NirC was amplified by polymerase chain reaction from genomic DNA extracted from *P. aeruginosa* PA14, omitting the signal peptide for periplasmic export as determined by *SignalP*5.0 (Almagro Armenteros *et al.*, 2019 ▸). The fragment was cloned between the NotI/KpnI restriction sites of a modified pCOLADuet plasmid (pVP008-ompA), yielding an expression vector that produces NirC with an N-terminal OmpA signal peptide for periplasmic export in *Escherichia coli* followed by a Tobacco etch virus (TEV) protease-cleavable StrepII-tag. Residue Glu71 was mutated to alanine by QuikChange Mutagenesis for the reasons outlined below. The success of cloning and mutagenesis was verified by the Eurofins Genomics sequencing service.

*E. coli* C43(DE3) cells (Miroux & Walker, 1996 ▸) were co-transformed with the NirC expression plasmid and with pEC86, a plasmid containing the cytochrome *c* maturation system of *E. coli*, which is necessary for the covalent attachment of heme to a suitable polypetide chain (Arslan *et al.*, 1998 ▸). LB medium (8 × 1 l) supplemented with 34 mg l^−1^ chloramphenicol, 30 mg l^−1^ kanamycin, 200 µ*M* δ-amino­levulinic acid and 12.5 µ*M* iron(II) sulfate was inoculated with 10 ml overnight culture and cultivated at 37°C with mild shaking until an optical density at 600 nm of between 0.6 and 0.8 was reached. The cultures were then induced with 1 m*M* isopropyl β-d-1-thiogalactopyranoside and further incubated at 20°C for 20 h. The cells were harvested by centrifugation and the pellets were stored at −20°C until needed.

The cell pellets were thawed, resuspended in lysis buffer (50 m*M* Tris–HCl pH 8.0, 150 m*M* NaCl) supplemented with protease inhibitors (cOmplete mini EDTA-free) and lysed by sonication. The lysate was centrifuged for 30 min at 36 000*g* followed by an additional centrifugation of the supernatant for 60 min at 100 000*g* to remove cell debris.

The cleared cell lysate was loaded onto a StrepTactin HC column (IBA, Göttingen, Germany) equilibrated with protein buffer (10 m*M* Tris–HCl pH 8.0, 150 m*M* NaCl) and connected to an ÄKTApurifier FPLC system (GE Healthcare, Boston, USA). The column was washed with five column volumes and subsequently eluted with three column volumes of protein buffer containing 5 m*M* d-desthiobiotin. Red colouration of elution fractions and UV–Vis absorption at 410 nm confirmed the presence of heme. Fractions containing no impurities as judged by SDS–PAGE analysis were pooled and digested with TEV protease at 4°C overnight with simultaneous dialysis against protein buffer. To remove undigested protein, the resulting solution was again loaded onto the StrepTactin column and the flowthrough was collected. As a final purification step, NirC was subjected to size-exclusion chromatography using a HiLoad 16/60 Superdex 75 column (GE Healthcare, Boston, USA). The pure protein was concentrated to 40 mg ml^−1^ with Vivaspin 20 (molecular-mass cutoff 3000) ultrafiltration units (Sartorius AG, Göttingen, Germany) and the concentration was determined spectrophotometrically using a NanoDrop 2000 (Thermo Fisher Scientific, Waltham, USA) and a calculated extinction coefficient of ∊_280_ = 11 m*M* ^−1^ cm^−1^. Macromolecule-production information is summarized in Table 1 ▸.

### 2.2. Crystallization   

Crystallization experiments were set up using a HoneyBee 961 pipetting robot (Digilab Genomic Solutions, Hopkinton, USA) and were monitored with a Rock Imager 1000 automated microscope (Formulatrix, Bedford, USA). Optimization screens were prepared utilizing a Formulator pipetting robot (Formulatrix, Bedford, USA). Experiments to crystallize wild-type NirC never yielded well diffracting crystals. Therefore, a mutation (E71A) suggested by the surface-entropy reduction server (Goldschmidt *et al.*, 2007 ▸) was introduced, leading to crystal growth in a few conditions of The Cryos Suite sparse-matrix screen (Qiagen, Hilden, Germany). The respective precipitants were further optimized by varying the concentration of precipitant and salts as well as the pH. Crystals usually began to appear after one week and took three more weeks to grow to full size (50–100 µm; Supplementary Fig. S1). The crystals were harvested and directly flash-cooled in liquid nitrogen without the addition of cryoprotectants. Crystallization information is summarized in Table 2 ▸.

### 2.3. Data collection and processing   

Data were collected on beamline P11 of the PETRA III synchrotron, DESY, Hamburg, Germany (Burkhardt *et al.*, 2016 ▸). The anomalous data set was processed using *DIALS* (Winter *et al.*, 2018 ▸), *POINTLESS* (Evans, 2011 ▸) and *AIMLESS* (Evans & Murshudov, 2013 ▸) from the *CCP*4 suite (Winn *et al.*, 2011 ▸). Despite the low completeness in the highest resolution shell, the data set was sufficient to obtain initial phases via SAD phasing. The data set used for final refinement was processed using *autoPROC* (Vonrhein *et al.*, 2011 ▸) executing *XDS* (Kabsch, 2010 ▸), *POINTLESS* (Evans, 2011 ▸), *AIMLESS* (Evans & Murshudov, 2013 ▸) and *STARANISO* (Tickle *et al.*, 2018 ▸). Anisotropic data truncation was applied assuming a local *I*/σ(*I*) of 1.2 to include useable data beyond the spherical resolution of 2.83 Å. The extent of the anisotropy is visualized in Supplementary Fig. S5. Data-collection and processing statistics are summarized in Table 3 ▸.

### 2.4. Structure solution and refinement   

The structure was solved by SAD phasing with anomalous differences arising from the heme Fe atoms using the *CRANK*2 phasing pipeline (Skubák & Pannu, 2013 ▸) executing *SHELXC*/*D* (Sheldrick, 2015 ▸) for substructure determination, *Parrot* (Cowtan, 2010 ▸) for density modification and *Buccaneer* (Cowtan, 2006 ▸) for model building. The iron substructure determination showed a decrease in occupancy for the 12th atom and beyond, hinting at the presence of 11 chains in the asymmetric unit. Automated model building produced a model consisting of 940 amino acids with an *R*_work_ of 35%. Inspection of the electron density showed that not all of the 11 chains were built correctly, requiring manual adjustments in *Coot* (Emsley & Cowtan, 2004 ▸) and further refinement in *REFMAC*5 (Murshudov *et al.*, 2011 ▸) with NCS restraints enabled. Final refinement was performed against the higher resolution native data set using *phenix.refine* (Afonine *et al.*, 2012 ▸) while lifting the applied NCS restraints, adding water molecules and riding H atoms, and applying TLS refinement. Depictions of the structure were made with *PyMOL* (version 1.8; Schrödinger). Refinement statistics are summarized in Table 4 ▸.

### 2.5. Size-exclusion chromatography coupled to a multi-angle laser light detector   

To assess the molecular mass of NirC, the isolated and concentrated protein solution (40 mg ml^−1^) was subjected to SEC-MALS experiments utilizing an Agilent Technologies 1260 Infinity II HPLC system (Santa Clara, USA) equipped with a Wyatt Optilab rEX diffraction-index detector and a miniDAWN TREOS II multi-angle laser light-scattering detector (Wyatt, Santa Barbara, USA). Separation was achieved with a Superdex 75 Increase 10/300 GL column (GE Healthcare, Boston, USA) with protein buffer as the eluent. To calculate the molecular mass, a d*n*/d*c* of 0.195 ml g^−1^ was assumed.

### 2.6. Structural bioinformatics   

Functional orthologs of NirC were identified by extracting orthologs from the OMA database (Altenhoff *et al.*, 2018 ▸) followed by manually filtering for the presence of the genes encoding the nitrite reductase NirS and the heme *d*_1_-biosynthesis enzyme NirF in their genetic neighbourhood. After aligning the functional orthologs with *Clustal Omega* (Ashkenazy *et al.*, 2016 ▸), sequence conservation was mapped onto the NirC monomer structure utilizing the *ConSurf* server (Fig. 3 and Supplementary Fig. S2; Sievers *et al.*, 2011 ▸). Structural homologs were identified with the *DALI* server (Holm & Laakso, 2016 ▸).

## 3. Results and discussion   

### 3.1. The simultaneous presence of NirC monomers and dimers in the asymmetric unit   

**Figure 2:**
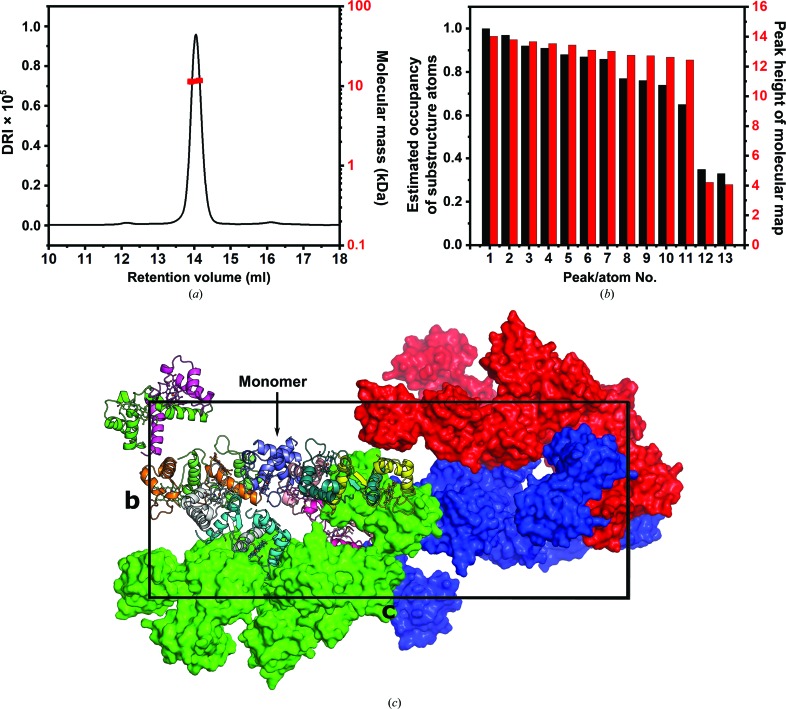
(*a*) The SEC-MALS chromatogram. The bar diagrams in (*b*) show the occupancies of atoms of the initially determined substructure as calculated with *SHELXD* (Sheldrick, 2015 ▸) and the height of the anomalous map peak calculated with phases of the final structure by *ANODE* (Thorn & Sheldrick, 2011 ▸). The drop after the 11th site indicates that only 11 Fe atoms are present. (*c*) The unit cell. One asymmetric unit is shown as a cartoon representation of the peptide backbone, with the covalently attached heme shown as as a ball-and-stick model and each chain coloured individually. The other asymmetric units are depicted as surface models coloured red, blue and green.

Approximately 2 mg of pure heme-bound NirC per litre of *E. coli* culture could reliably be obtained using the procedure described above. Size-exclusion chromatography as well as multi-angle light scattering suggested that the concentrated protein solution used in crystallization trials only contains monomers with a molecular mass of 11.5 kDa ± 0.9% (10.5 kDa expected; Fig. 2 ▸). Because the wild-type protein failed to crystallize, we attempted to reduce the surface entropy by mutating Glu71 to alanine, finally yielding cube-shaped crystals that diffracted anisotropically and possessed a very large orthorhombic unit cell (Supplementary Fig. S1). To obtain the initial phases, we made use of the presence of anomalous differences in diffraction data collected at the Fe *K* edge, leading to the positioning of 11 Fe atoms. Assuming that NirC contains one Fe atom bound to the covalently attached heme, this suggests a solvent content of 58%. A combination of automated model building and manual adjustments led to a final model with *R*_work_ = 21.1% and *R*_free_ = 24.7% that indeed consisted of 11 chains in the asymmetric unit. Interestingly, despite the use of monomeric NirC for crystallization, ten of these chains form dimers by exchanging their N-terminal α-helices, as recognizable by different traces in the 2*F*_o_ − *F*_c_ density for the ‘hinge loop’ (Gly25–Gly33; Fig. 5). The dimers fall into two different types, as outlined below. Surprisingly, the 11th chain is monomeric, *i.e.* the crystal form obtained here contains both dimers and monomers of NirC at the same time. Analysis of the crystal packing reveals that the surface-entropy-reducing mutation E71A enables packing interactions that are not possible with the glutamate side chain. An example is shown in Supplementary Fig. S6. Importantly, the mutation is not part of the dimer interfaces or the hinge loop, suggesting that it does not cause the simultaneous presence of different NirC oligomeric states observed here.

The formation of dimers from monomeric *c*-type cytochromes is a well known phenomenon (Hirota, 2019 ▸). It has been shown that the folding of horse cytochrome *c* starts with hydrophobic interactions between the N- and C-terminal helices, yielding 3D domain-swapped oligomers if the helices belong to different chains (Parui *et al.*, 2013 ▸). Therefore, denaturation and subsequent refolding at high protein concentration is often utilized to produce such dimers (Hayashi *et al.*, 2012 ▸; Nagao *et al.*, 2015 ▸). Apparently, the high NirC concentration and the presence of precipitants in the crystallization experiments performed here are sufficient to allow an equilibrium between dimers and monomers.

Whereas 3D domain swapping is often observed in crystal structures (reviewed in Liu & Eisenberg, 2002 ▸), the simultaneous presence of different homooligomeric states of one protein in the same asymmetric unit is extremely rare and we are aware of only two other unambiguous examples, namely the FeSII protein from *Azotobacter vinelandii* (PDB entry 5frt; B. V. Kabasakal, C. A. R. Cotton, L. Lieber & J. W. Murray, unpublished work) and an engineered tenascin protein (PDB entry 2rbl; Hu *et al.*, 2007 ▸). In both cases the asymmetric unit was found to contain monomers and 3D domain-swapped dimers, similar to the crystal structure of NirC reported here. In addition, there may be less obvious instances that do not involve 3D domain swapping, such as a non-oligomerizing variant of the auxin response factor from *Arabidopsis thaliana* (PDB entry 4nj7). This protein crystallized with 16 chains in the asymmetric unit, which may be regarded as dimers and pentamers (Korasick *et al.*, 2014 ▸), although it also seems possible to explain the packing by assuming only monomers.

### 3.2. NirC shows high similarity to other monoheme *c*-type cytochrome domains encoded by the *nir* operon   

**Figure 3:**
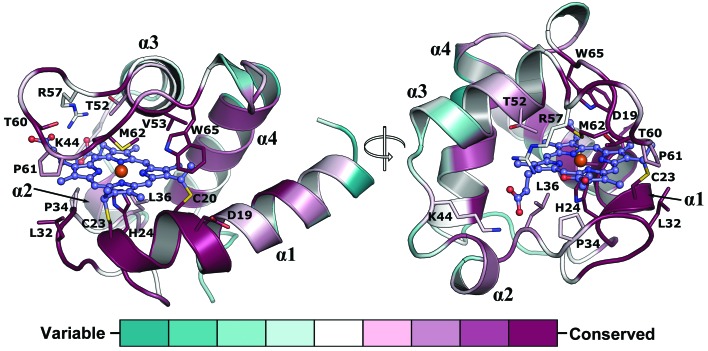
The NirC monomer shown as a cartoon with residues at a maximum distance of 4 Å from the covalently attached heme presented as sticks. Colours are based on sequence conservation as defined by *ConSurf* (Ashkenazy *et al.*, 2016 ▸). The sequence alignment used to determine conservation is shown in Supplementary Fig. S3.

**Figure 4:**
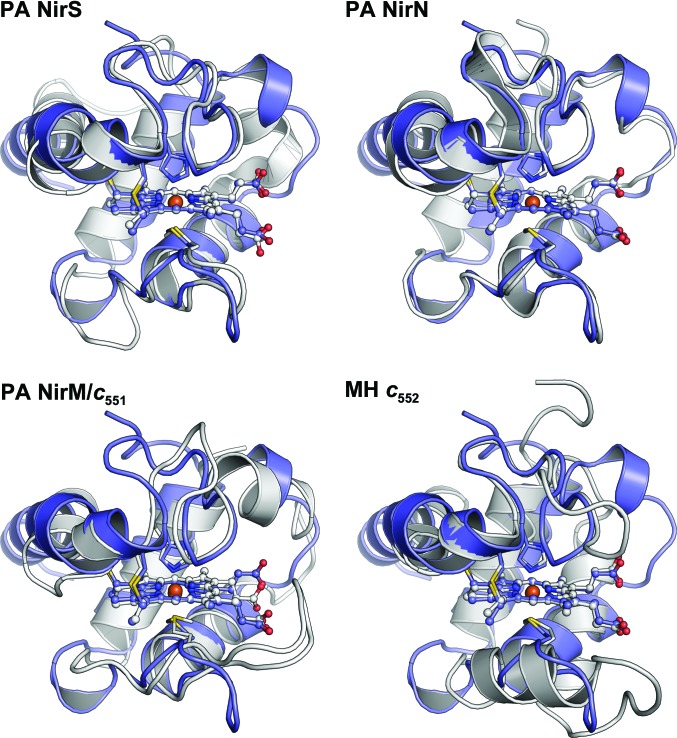
Comparison of the structure of monomeric NirC with the cytochrome *c* domains of NirS and NirN as well as with NirM from *P. aeruginosa* (PA) and cytochrome *c*_552_ from *Marinobacter hydrocarbonoclasticus* (MH) after superposition of the covalently attached heme. All structures are depicted as cartoons and the covalently attached heme is shown as a ball-and-stick model. NirC is shown in blue and the compared structure is in white.

NirC displays a typical monoheme *c*-type cytochrome fold containing three α-helices (α1, α3 and α4) that surround a thioether-bound heme *c* moiety (Fig. 3 ▸). The iron cation is coordinated by His24 and Met62, which is consistent with mutagenesis studies performed using the orthologue from *P. pantotrophus* (Zajicek *et al.*, 2009 ▸). The heme-binding crevice consists of a predominantly hydrophobic pocket established by Leu32, Pro34, Leu36, Thr52, Val53, Thr60, Pro61 and Trp65, which are also highly conserved in NirC proteins from other species. In *P. aeruginosa* NirC, the propionate groups of heme form hydrogen bonds to Lys44 and Arg57, two residues that are replaced by hydrophobic amino acids in some species, probably reflecting the fact that the heme propionate groups are solvent-exposed and do not require stabilizing interactions with the protein to be accommodated. Sequence alignment reveals that in NirC the heme-attachment motif (C*XX*CH) is always preceded by a conserved aspartate (Asp19), the side chain of which is solvent-exposed, and followed by a conserved GGLG motif residing on a loop connecting helices α2 and α3 (Supplementary Fig. S3). To­gether with residues in the neighbourhood of the iron-ligating Met62, these motifs create a conserved surface patch, which may be required to form an interaction site for other proteins such as, for example, the heme *d*_1_-biosynthesis protein NirF, the function of which is not clear but may lie in an oxido­reduction within the last steps of the pathway (Klünemann *et al.*, 2020 ▸). Alternative hypotheses suggest involvement as an electron transporter of the nitrite reductase NirS (Hasegawa *et al.*, 2001 ▸). A role in heme *d*_1_ biosynthesis itself, on the other hand, seems to be supported by the finding that similarity searches with *DALI* (Holm & Laakso, 2016 ▸) identify the cytochrome *c* domains of the *nir*-operon proteins nitrite reductase (NirS) and the heme *d*_1_-biosynthesis enzyme NirN as most similar to NirC, while NirM and cytochrome *c*_552_, the known electron transporters that interact with NirS in nitrite reduction, differ to a larger extent (Fig. 4 ▸ and Supplementary Table S1). In NirN and NirS, the cytochrome *c* domains are covalently attached to a β-propeller domain and function as electron shuttles in the reactions catalysed by these enzymes. This suggests that NirC may perform a similar function with NirF, which is a standalone β-propeller protein with high similarity to the β-propeller domains of NirN and NirS. Interestingly, superposition of NirC and NirF onto the respective domains of NirS leads to a complex in which the conserved surface patch of NirC is oriented towards the propeller of NirF, and Leu32 of the conserved GGLG motif is buried in a hydrophobic pocket (Supplementary Fig. S4). This may further corroborate the involvement of NirC in heme *d*_1_ biosynthesis.

### 3.3. NirC forms two different types of dimers   

The dimers of NirC found here form by exchanging the N-terminal helix (Pro4–His24), which results in heme being coordinated by His24 and Met62 from two different chains, similar to what has been observed for dimers of cytochrome *c*_551_ (NirM) from *P. aeruginosa* and cytochrome *c*_552_ from *Hydrogenobacter thermo­philus* (HT) (Hayashi *et al.*, 2012 ▸; Nagao *et al.*, 2015 ▸). Notably, dimerization does not have a major impact on the structure of the heme-binding site, indicating that monomers and dimers could display similar biochemical properties, as has already been found for other N-terminally domain-swapped cytochromes (Nagao *et al.*, 2015 ▸; Hayashi *et al.*, 2012 ▸).

**Figure 5:**
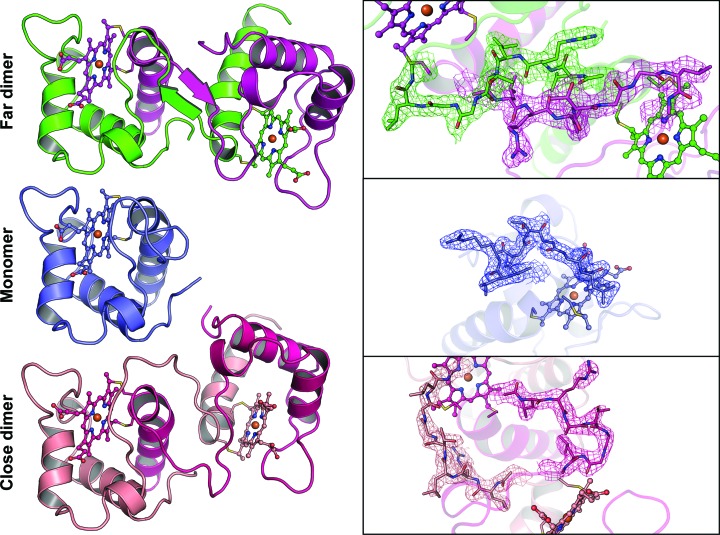
Depiction of the two different oligomerization states found in the crystal structure of NirC. On the left, the NirC monomer and both dimer conformations are shown as described in Fig. 1 ▸ after the superposition of one protomer. The backbone of NirC is shown as a cartoon with the covalently attached heme as a ball-and-stick model. On the right, the residues of the hinge loop are depicted as stick models with a 2*F*_o_ − *F*_c_ map at a σ level of 1, coloured according to the associated chain and individually orientated to allow an unobstructed view.

Closer inspection reveals that the five dimers present in the asymmetric unit fall into two groups that are distinguishable by heme iron distance, internal symmetry and the relative position of the hinge loop (25-GLRLTGGLG-33; Fig. 5 ▸). In the three ‘far dimers’ the average iron–iron distance is 28.5 ± 0.2 Å, whereas it is 24.9 ± 0.1 Å in the two ‘close dimers’. Interestingly, the two dimeric forms of NirC display different amounts of internal symmetry, as can be quantified by the ‘GloA-score’. Naturally occurring homodimers are usually highly symmetric, with GloA-scores under 0.4 Å, while scores above 3 Å are indicative of substantial asymmetry (Swapna *et al.*, 2012 ▸). In the dimers of NirC observed here, the GloA-score is 0.9 ± 0.2 Å for the far dimers, compared with 3.05 ± 0.01 Å for the close dimers; this is indicative of substantial asymmetry in the latter, which is readily discernible after the superposition of one protomer of a dimer onto the other (Supplementary Fig. S2). The basis for the formation of different dimers in NirC seems to be rooted in the hinge loop, which with nine residues is much longer and allows more flexibility than the three residues found in *P. aeruginosa* cytochrome *c*_551_ or HT cytochrome *c*_552_ (Hayashi *et al.*, 2012 ▸; Nagao *et al.*, 2015 ▸). The importance of the length and hence the flexibility of the hinge loop is reflected in the observation that its elongation in HT cytochrome *c*_552_ induced different N- and C-terminally domain-swapped dimers (Ren *et al.*, 2015 ▸). In NirC, the increased flexibility leads to antiparallel β-sheet-like interactions of Leu26, Arg27 and Leu28 in the far dimers and no interactions, but with elevated *B* factors of the hinge loop in the close dimers.

The relevance of dimer formation in NirC and the associated different levels of asymmetry are not clear at present. Previous work with eukaryotic monoheme *c*-type cytochromes suggested that 3D-domain-swapped oligomers are involved in apoptosis (Junedi *et al.*, 2014 ▸), which seems to be in line with the finding that several other 3D-domain-swapped proteins have a regulatory function. Examples include bovine RNase, where only the dimer displayed toxicity towards tumour cells (Di Donato *et al.*, 1995 ▸), and the regulatory domain of α-isopropylmalate synthase from *Mycobacterium tuberculosis*, which forms via 3D domain swapping (Koon *et al.*, 2004 ▸). Because dimer formation of NirC is clearly linked to the hinge loop and because this loop is highly conserved amongst NirC homologues from different species, it is possible that dimers of NirC also have a regulatory role. However, the fact that we did not detect dimers even in highly concentrated solutions, together with the observation that crystallization took relatively long and required the introduction of a surface-entropy-reduction mutant that enabled crystal formation through new crystal contacts, currently argues for a crystallization artefact that points towards structural malleability of these proteins rather than a physiological importance of NirC dimers.

In summary, our work provides new insight into NirC, a monoheme *c*-type cytochrome that is likely to be involved in the biosynthesis of heme *d*_1_, an essential cofactor in nitrite reduction by denitrifying bacteria such as the opportunistic pathogen *P. aeruginosa*. While the simultaneous observation of NirC monomers and dimers in the same asymmetric unit of the crystal form obtained here may reiterate typical properties of this protein family, the mapping of conserved sequence motifs onto the structure of the monomer may offer new avenues to decipher the function of NirC.

## 4. Related literature   

The following references are cited in the supporting information for this article: Brown *et al.* (1999 ▸), Klünemann *et al.* (2019 ▸), Matsuura *et al.* (1982 ▸), Nurizzo *et al.* (1998 ▸) and Robert & Gouet (2014 ▸).

## Supplementary Material

PDB reference: NirC, 6tp9

Supplementary Table and Figures. DOI: 10.1107/S2059798320003101/ud5014sup1.pdf

## Figures and Tables

**Table 1:** Macromolecule-production information

Source organism	*P. aeruginosa*
Cloning primer†	AAAGCGGCCGCGACGAGCATCCCGATGCCC
	AAAGGTACCTCATGGGGCGATCTCTCCTTCG
Mutagenesis primer	GCCTGCTCAGCGCAGACGACGCCGG
	CCGGCGTCGTCTGCGCTGAGCAGGC
Cloning vector	pVP008-ompA modified from pCOLADuet
Expression vector	pVP008-ompA-NirC-E71A
Expression host	*E. coli* C43(DE3) (Miroux & Walker, 1996 ▸)
Complete amino-acid sequence of the recombinant protein‡	*MKKTAIAIAVALAGFATVAQ*AMAS**WSHPQFEK**VDENLYFQGGGRDEHPDARRQAQLRHLLLQDCGSCHGLRLTGGLGPALTPEALRGKPRESLVATVLMGRPQTPMPPWAGLLS**A**DDAGWLVDRLIEGEIAP

† The NotI/KpnI restriction sites are underlined.

‡ The TEV protease cleavage site is underlined, the StrepII-tag is in bold, the OmpA leader peptide is in italics and the E71A mutation is in bold and underlined.

**Table 2:** Crystallization

	Crystal used for SAD phasing	Crystal used for final structure refinement
Method	Sitting-drop vapour diffusion	Sitting-drop vapour diffusion
Plate type	Intelli-Plate 96-3	Intelli-Plate 96-3
Temperature (K)	293	293
Protein concentration (mg ml^−1^)	27	40
Buffer composition of protein solution	10 m*M* Tris–HCl pH 8.0, 150 m*M* NaCl	10 m*M* Tris–HCl pH 8.0, 150 m*M* NaCl
Composition of reservoir solution	15% glycerol, 8.5 m*M* NiCl_2_, 85 m*M* Tris–HCl pH 8.5, 17% PEG MME 2000	15.33% glycerol, 0.1 *M* MES–NaOH pH 6.3, 10.8% PEG 20 000
Volume and ratio of drop	200 nl:200 nl	200 nl:200 nl
Volume of reservoir (µl)	60	60

**Table 3:** Data collection and processing Values in parentheses are for the highest resolution shell.

	Anomalous data set	Native data set
Diffraction source	Beamline P11, PETRA III	Beamline P11, PETRA III
Wavelength (Å)	1.738	1.033
Temperature (K)	100	100
Detector	PILATUS 6M	PILATUS 6M
Crystal-to-detector distance (mm)	544	457.5
Rotation range per image (°)	0.1	0.1
Total rotation range (°)	1080	360
Exposure time per image (s)	0.1	0.04
Space group	*P*2_1_2_1_2_1_	*P*2_1_2_1_2_1_
*a*, *b*, *c* (Å)	77.7, 80.6, 200.0	76.8, 80.2, 195.7
α, β, γ (°)	90, 90, 90	90, 90, 90
Resolution range (Å)	200–3.4 (3.7–3.4)	spherical: 99.2–2.8 (2.9–2.8)
		ellipsoidal: 99.16–2.19 (2.47–2.19)
Diffraction limit along principal axes of fitted ellipsoid	—	2.98 (a*), 2.91 (b*), 2.12 (c*)
Total No. of reflections	368556 (6018)	460561 (18799)
No. of unique reflections	13722 (873)	35413 (1772)
Completeness, spherical (%)	77.3 (24.9)	53.6 (8.9)
Completeness, ellipsoidal (%)	—	92.7 (74.8)
Multiplicity	26.9 (6.9)	13.0 (10.6)
〈*I*/σ(*I*)〉	10.5 (2.2)	10.9 (1.8)
CC_1/2_	0.993 (0.721)	0.998 (0.610)
*R*_p.i.m._	0.046 (0.404)	0.058 (0.416)
Overall *B* factor from Wilson plot (Å^2^)	63	23

**Table 4:** Structure solution and refinement Values in parentheses are for the highest resolution shell.

Resolution range (Å)	56.31–2.19 (2.25–2.19)
No. of reflections, working set	35402 (93)
No. of reflections, test set	1746 (9)
Final *R*_cryst_	0.211 (0.389)
Final *R*_free_	0.247 (0.756)
No. of non-H atoms	
Protein	6879
Ligand	473
Water	154
Total	7506
R.m.s. deviations	
Bond lengths (Å)	0.004
Angles (°)	0.71
Average *B* factors (Å^2^)	
Overall	32
Protein	33
Ligand	27
Water	29
Ramachandran plot	
Most favoured (%)	97.44
Allowed (%)	2.56
