# Supplementary material for: The crystal structure of the heme d 1 biosynthesis-associated small c-type cytochrome NirC reveals mixed oligomeric states in crystallo
Source: Acta Crystallogr D Struct Biol. 2020 Mar 25;76(Pt 4):375–84. doi: 10.1107/S2059798320003101 (PMC7137109; doi:10.1107/S2059798320003101)
Supplement: Supplementary file 1 [file d-76-00375-sup1.pdf]

STRUCTURAL BIOLOGY

Volume 76 (2020)

Supporting information for article:

The crystal structure of the heme *d*1 biosynthesis-associated small *c*-type cytochrome NirC reveals mixed oligomeric states *in crystallo*

Thomas Klünemann, Steffi Henke and Wulf Blankenfeldt

Table S1 Selected high scoring results of the Dali server discussed in this publication.

Organism and Name	PDB-ID	Z-score	lali	rmsd (Å)	%id	Citation
PA NirN	6rtd	12.2	76	1.6	37	(Klünemann *et al.*, 2019)
PA NirS	1nno	12	81	2.3	26	(Nurizzo *et al.*, 1998)
PA NirM/ cyt c551	351c	7.8	71	2.5	25	(Matsuura *et al.*, 1982)
MH cyt c552	1cno	5.2	65	2.7	23	(Brown *et al.*, 1999)

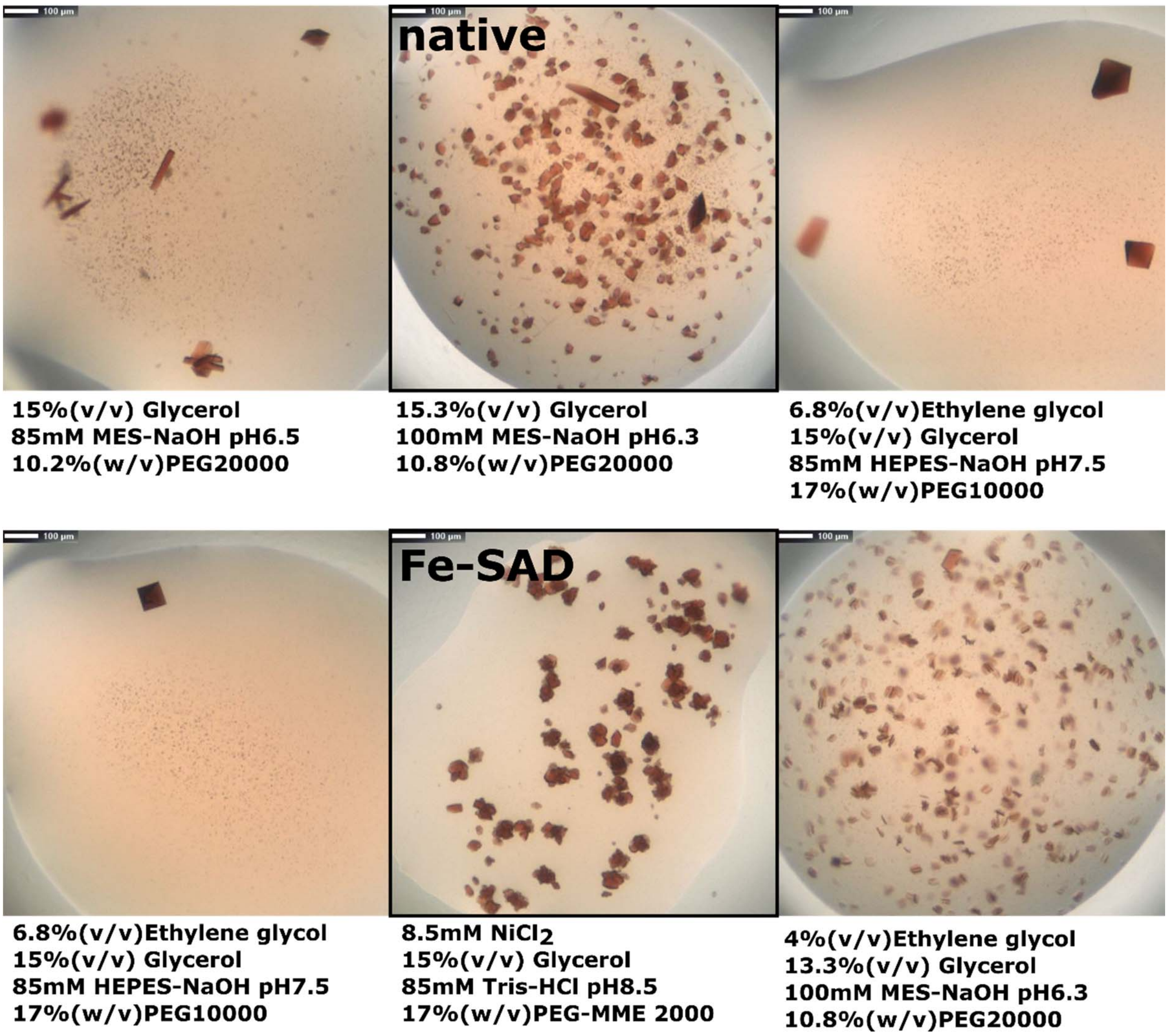

Figure S1 Crystals of NirC^{E71A} grown in different conditions used for diffraction experiments. Conditions used in this study are indicated by a black frame.

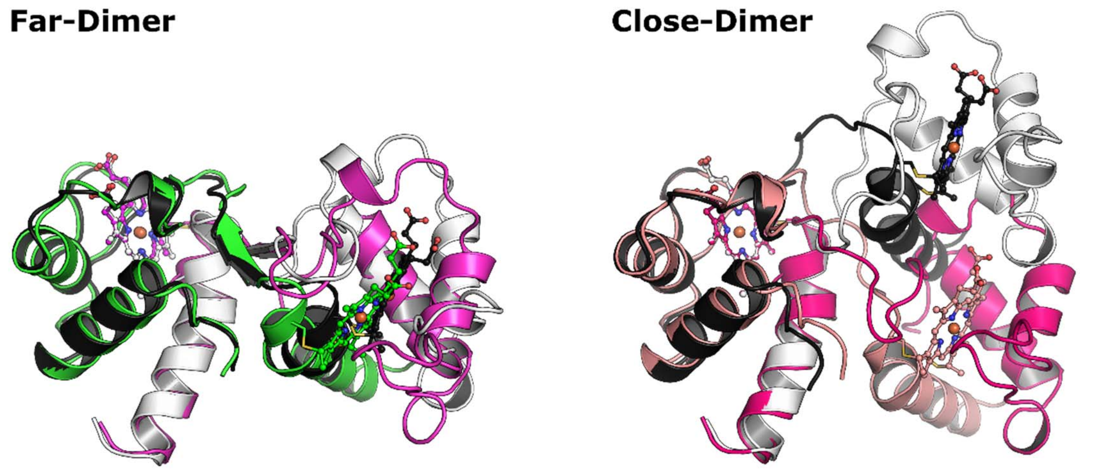

Figure S2 Depiction of NirC dimers. To highlight the asymmetry, one protomer was superposed with the copy of the opposite one. Except for the colouration of the copied dimer (black and white), the colour and representation of the model is the same as lied out in Figure 5 in the main text.

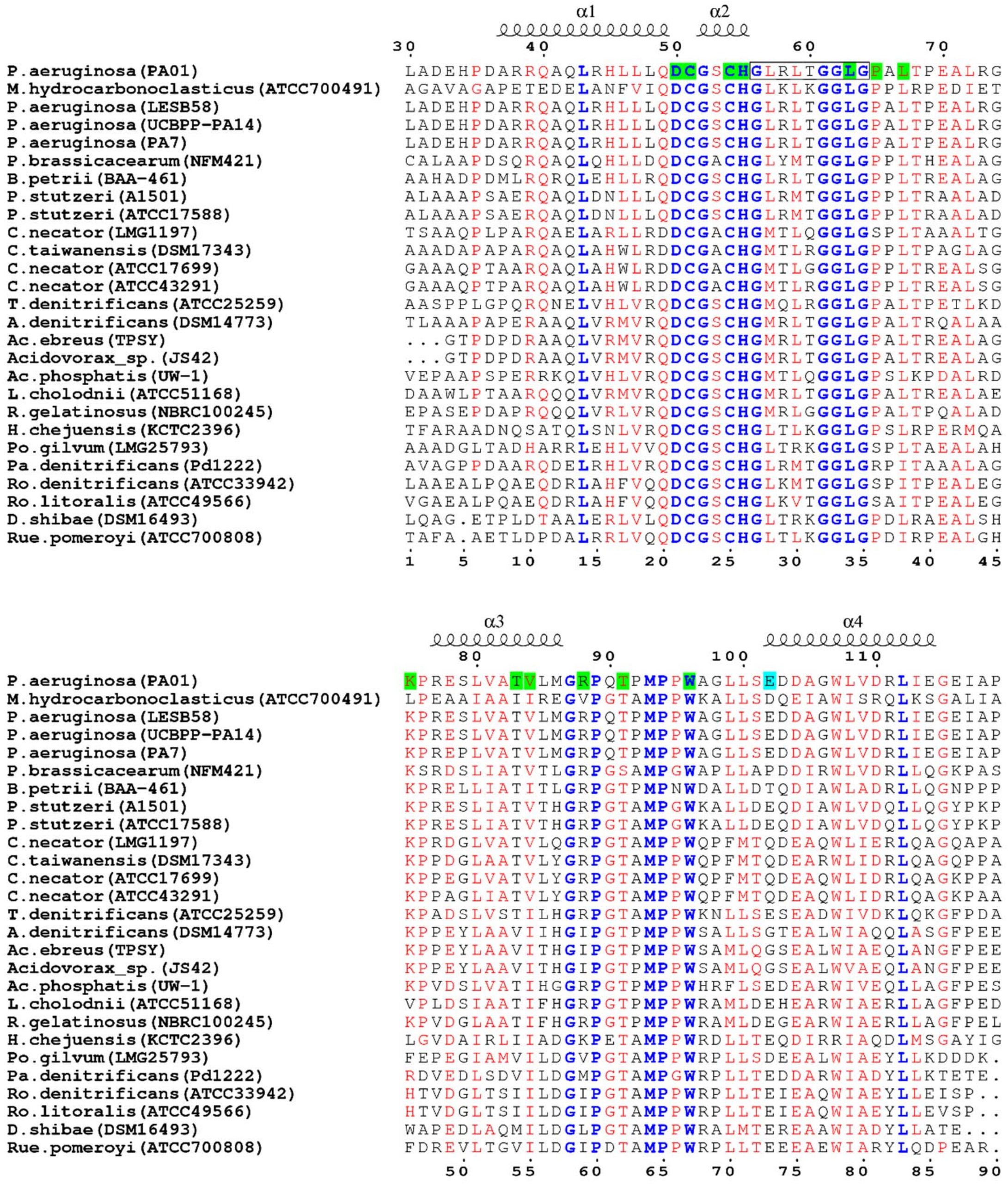

Figure S3 Sequence alignment of NirC orthologs from different denitrifying species extracted from the OMA server (Altenhoff *et al.*, 2018). Green boxes mark heme-interacting residues. A cyan box indicates E71 mutated to alanine to enable crystal formation and the black box highlights residues belonging to the hinge loop, which adopts different conformation in the 3D domain swapped dimers. Bold blue letters indicate identity in all residues and red letters are equivalent to 70%. Sequence numbering is based on whole protein including the signal peptide. The figure was prepared with ESPript3.0 (Robert & Gouet, 2014).

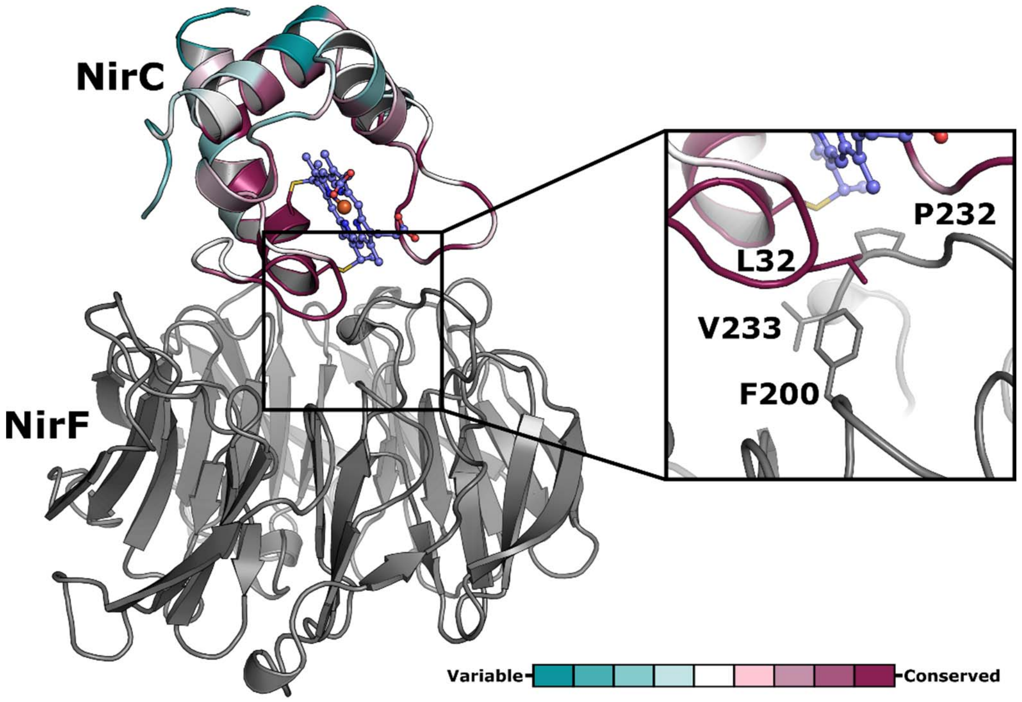

Figure S4 Depiction of NirC (coloured according to sequence conservation) and NirF (grey) (PDB: 6TV2) after superposition onto the cytochrome *c* and d_1-domains of nitrite reductase NirS (PDB: 1nir), respectively.

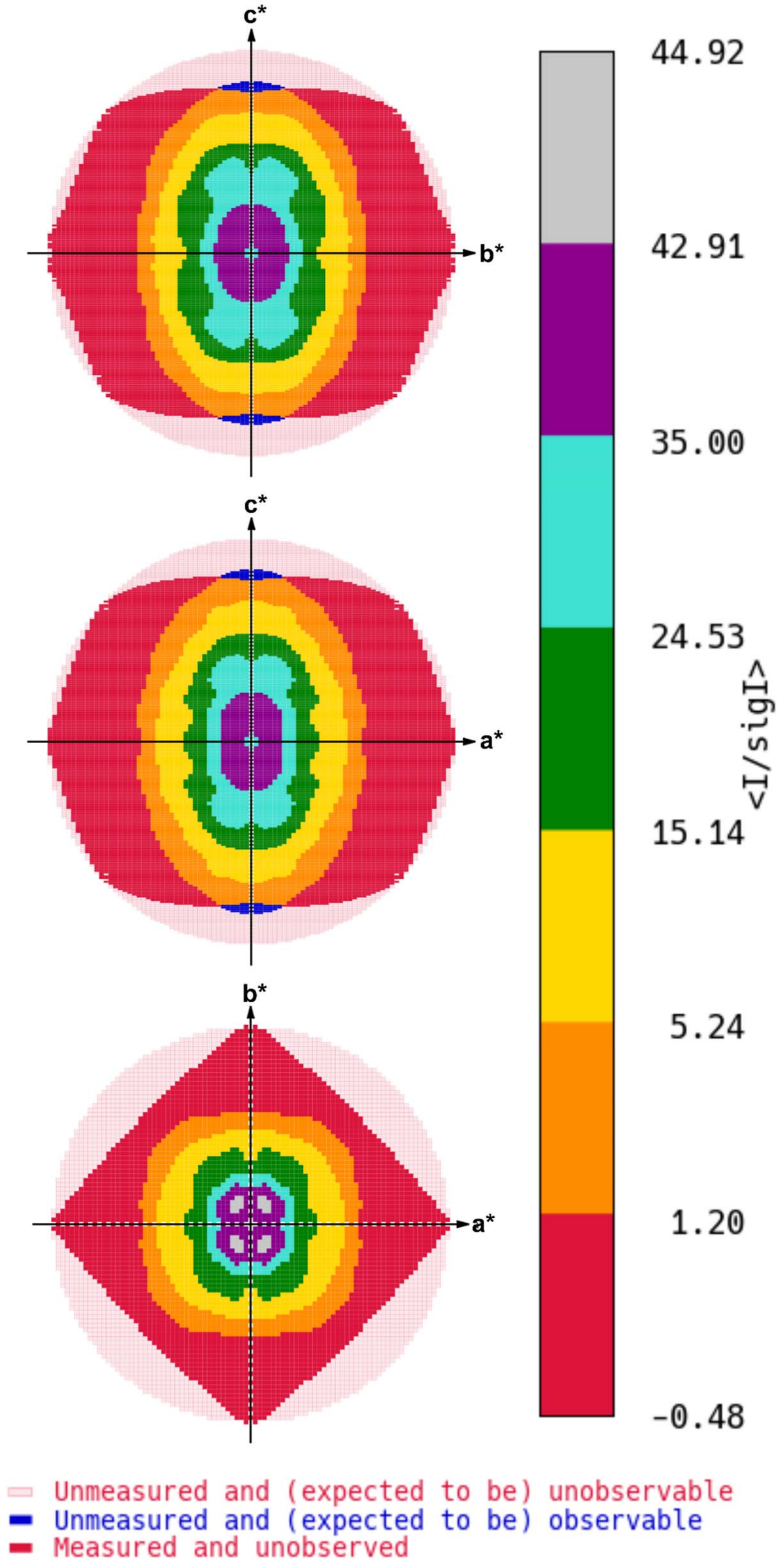

Figure S5 Graphical representation of the <I/σ(I)>-values along the principal axes of the reciprocal lattice.

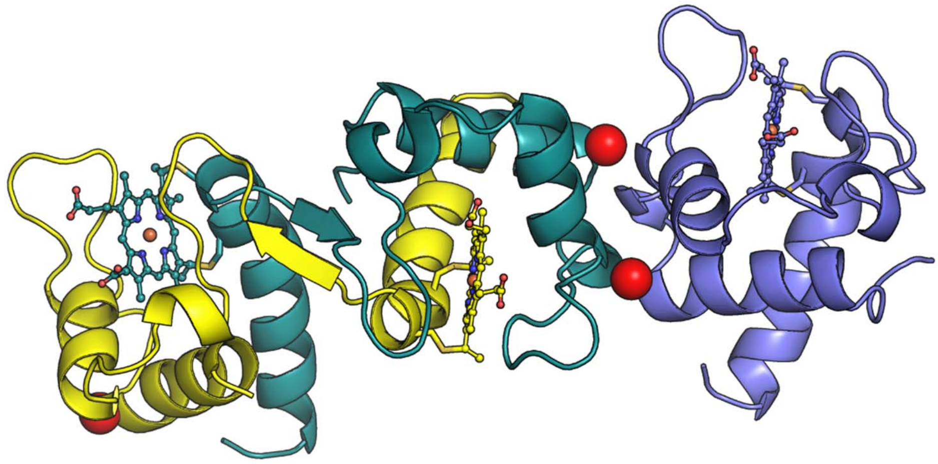

Figure S6 Depiction of chain G (blue), J (teal) and D (yellow) with a red sphere highlighting the mutation site of E71A, which was introduced to increase the crystallisability of NirC.
